# Alterations of Renal Function in Patients with Diabetic Kidney Disease: A BOLD and DTI Study

**DOI:** 10.1155/2022/6844102

**Published:** 2022-09-30

**Authors:** Xiaobao Wei, Runyue Hu, Xiaoli Zhou, Lihua Ni, Dongqing Zha, Huiling Feng, Haibo Xu, Xiaoyan Wu

**Affiliations:** ^1^Department of Nephrology, Zhongnan Hospital of Wuhan University, Wuhan, Hubei, China; ^2^Department of Radiology, Zhongnan Hospital of Wuhan University, Wuhan, Hubei, China

## Abstract

**Objectives:**

Our study aims to determine the patterns of renal oxygenation changes and microstructural changes by BOLD and DTI with deteriorating kidney function in patients with diabetic kidney disease (DKD).

**Methods:**

Seventy-two patients with type 2 diabetes mellitus (DM) and twenty healthy controls (HCs) underwent laboratory examinations, and renal BOLD and DTI images were obtained on a 3T-MRI machine. *R*2^*∗*^, fractional anisotropy (FA), and average diffusion coefficient (ADC) values were evaluated. DM patients were divided into three subgroups (Group-DI/DII/DIII, based on urinary albumin-creatinine ratio (UACR)) and a nondiabetic kidney disease group (Group-NDKD). D-value and MCR of *R*2^*∗*^ and FA were proposed to evaluate the differentiation between medulla and cortex of the individual kidney among HCs and three subgroups for reducing individual differences. Comparisons were made between NDKD and kidney function-matched DKD patients. Correlations between MRI parameters and renal clinical indices were analyzed.

**Results:**

Compared with Group-HC/DI, medullary *R*2^*∗*^ and FA values were significantly different in Group-DII/III. The D-value of *R*2^*∗*^ and FA in Group-III were significantly smaller than that in Group-HC. However, only MCR of *R*2^*∗*^ in Group-III was significantly smaller than that in HCs. Medullary *R*2^*∗*^ and FA were negatively associated with serum creatinine (SCr) and cystatin C (Cys C) and positively associated with eGFR.

**Conclusions:**

With renal function declining, BOLD and DTI could capture alterations including the first rising and then falling medullary *R*2^*∗*^, continuously declining medullary FA, and apparent cortex-medullary differentiation in DKD patients. The MRI parameters showed renal changes accompanied by varying degrees of albuminuria, sharing common involvement in DKD and NDKD patients, but it was hard to distinguish between them. BOLD seemed more sensitive than DTI in identifying renal cortex-medullary differentiation.

## 1. Introduction

Diabetes usually results in complications, affecting the nerves, eyes, kidneys, and cardiovascular system, which leads to decreased quality of life and a greater risk of death than similarly aged healthy people [1]. DKD has been reported to have an incidence of 20% ∼ 40% in diabetic patients in European and American countries [2, 3]. In 2011, the prevalence of DKD in hospitalized patients in China began to exceed that of glomerular nephritis-related chronic kidney disease and the gap has been increasing [4, 5].

The American Diabetes Association (ADA) and Kidney Disease: Improving Global Outcomes (KDIGO) group suggest that the detection of the urinary albumin/creatinine ratio (UACR) and eGFR should be appropriately frequent to better diagnose and treat DKD [6, 7]. The clinical symptoms of early DKD are not obvious. Patients may have dry mouth, anorexia, stomach upset, fatigue, weakness, mild eyelid edema, or even numbness, but usually do not have symptoms such as moderate edema, nausea, and vomiting. When urinary microalbumin (uAlb) or SCr is used as a basic means to predict and screen DKD, once it is abnormal, kidney function already declines significantly [8, 9]. Biomarkers, including indicators of glomerular damage and tubular damage, inflammatory factors, proteomics, and even microRNAs, require in-depth clinical validation [10]. Moreover, the renal lesions of DKD cannot be visually displayed by routine ultrasound, CT, and MRI examination.

It is essential to assess kidney damage in DKD patients via a noninvasive and intuitive method. Sufficient evidence indicates that chronic hypoxia and interstitial fibrosis in the kidneys are involved in the occurrence and development of DKD [11–14]. The early pathological manifestations are increased glomerular volume, increased mesangial matrix, and thickened basement membrane, and the late stage is mostly sclerotic changes. Blood oxygenation level-dependent magnetic resonance imaging (BOLD-MRI) is the only noninvasive technology for evaluating the renal oxygen content in renal living tissue instead of invasively inserting microelectrodes into the kidneys directly [15]. In BOLD, deoxygenated hemoglobin is paramagnetic, and its content will affect the uniformity of the magnetic field in the surrounding local tissues, which is transformed into the *T*2^*∗*^ signal. Since *R*2^*∗*^ = 1/*T*2^*∗*^, anything that could reduce the concentration of deoxygenated hemoglobin decreases the *R*2^*∗*^ value. An increase in the *R*2^*∗*^ value corresponds to a decrease in oxygenated hemoglobin, a decrease in partial pressure of oxygen, and tissue hypoxia. The hyperperfusion of the renal medulla and its oxygen partial pressure gradient allow BOLD-MRI to detect slight fluctuations in deoxyhemoglobin in the renal medulla, making it easier to evaluate renal oxygenation. Diffusion tensor imaging (DTI) is the only noninvasive technology for evaluating water molecular diffusion in the renal living tissue, which can detect the altered structures in the medulla by abnormal diffusion of water molecules in patients with early DKD [16, 17]. Diffusion refers to the random and irregular movement of molecules, which is an important physiological activity of the human body. Renal DTI mainly describes the range of diffusion movement of water molecules in unit time by ADC and describes the proportion of anisotropic components of water molecules in the whole diffusion tensor by FA to reflect the renal microstructure. The purpose of our research was to explore the relationships between BOLD and DTI alterations and the renal function decline in DKD patients and to explore whether there is a difference between the renal damage exhibited by NDKD and DKD in DM.

## 2. Materials and Methods

### 2.1. Participants

This study was approved by the Ethics Committee. All participants signed an informed consent form before participating in the experiment. We recruited 80 patients from the Department of Nephrology and Endocrinology in our hospital and 20 healthy volunteers matched the sex and age criteria from December 2020 to June 2022. The patient's inclusion criteria were as follows: (1) age 18 to 80 years; (2) type II DKD, in accordance with the 2020 American Diabetes Association diagnosis, eGFR >30 ml/min/1.73 m^2^; (3) type II simple DM, no renal complications or biochemical abnormalities; (4) nondiabetic kidney disease (NDKD) patients (type II diabetes with CKD, e.g., hypertensive nephropathy, nephrotic syndrome, and tubulointerstitial disease), eGFR >30 ml/min/1.73 m^2^; (5) no serious complications of diabetes, such as diabetic ketosis, hyperglycemic hyperosmolarity syndrome, and hypoglycemia; no diuretic use within one month and no renal replacement therapy; and (6) no MRI contraindications. Eight cases were completely excluded because of multiple or large kidney cysts (the presence of a cyst larger than 4 cm in diameter in one kidney or the presence of multiple cysts with the sum of their diameters larger than 4 cm, *n* = 6) and eGFR <30 ml/min/1.73 m^2^ (eGFR too low, no control, *n* = 2). Finally, 62 T2DM and DKD patients, and 10 NDKD patients were included in this study. 62 patients were divided into three subgroup according to UACR: DI group (UACR ≤ 30 mg/g); DII group (30 < UACR ≤ 300 mg/g); DIII group (UACR> 300 mg/g), and NDKD group (30 ml/min/1.73 m^2^ < eGFR < 90 ml/min/1.73 m^2^).

Healthy volunteers were included accordant with the following specific criteria: no history of urinary system disease, no diabetes, and no use of any drugs that damage the kidneys; blood pressure control within 140/90 mmHg; no abnormalities in routine blood tests and liver and kidney function tests; good cooperation with MRI examinations; and no MRI contraindications.

### 2.2. Study Design

We used a 3.0-T machine (Signa 750w, GE Healthcare, USA) with a 16-channel body coil. Before the scans, all the subjects were abstained from consuming food and water for 4 hours and underwent breathing training. Routine *T*2 and *T*1 sequences were scanned after localization to exclude significant renal lesions.

### 2.3. Scanning Parameters

Coronal renal BOLD scanning: we used a multiple fast gradient recalled echo (MFGRE) sequence with 8 echoes with the following parameters: TR = 100 ms, TE = 3.3–26.2 ms, FOV = 32 cm × 32 cm, NEX = 1, slice thickness = 5 mm, spacing = 0.4 ms, matrix size = 256 × 256, bandwidth = 62.50 kHz per pixel, and flip angle = 20°. Images were acquired during a 14 second breath hold with an acquisition time of approximately 27 s, and the central line was placed at the renal hilum, with five layers in front and behind.

Coronal renal DTI scanning: images with respiratory triggering were acquired with a diffusion gradient oriented in 16 directions, b-values of (0 s/mm^2^, 600 s/mm^2^), using a spin-echo (SE) echo-planar imaging (EPI) sequence with the following parameters: TR = 2000 ms, TE = 66–462 ms, FOV = 36 cm × 32 cm, NEX = 1, slice thickness = 5.0 mm, spacing = 0 ms, matrix = 128 × 128, bandwidth = 250 kHz per pixel, acquisition time = 36–47 seconds, and a range covering both kidneys.

### 2.4. Image Analysis

**Figure 1:**
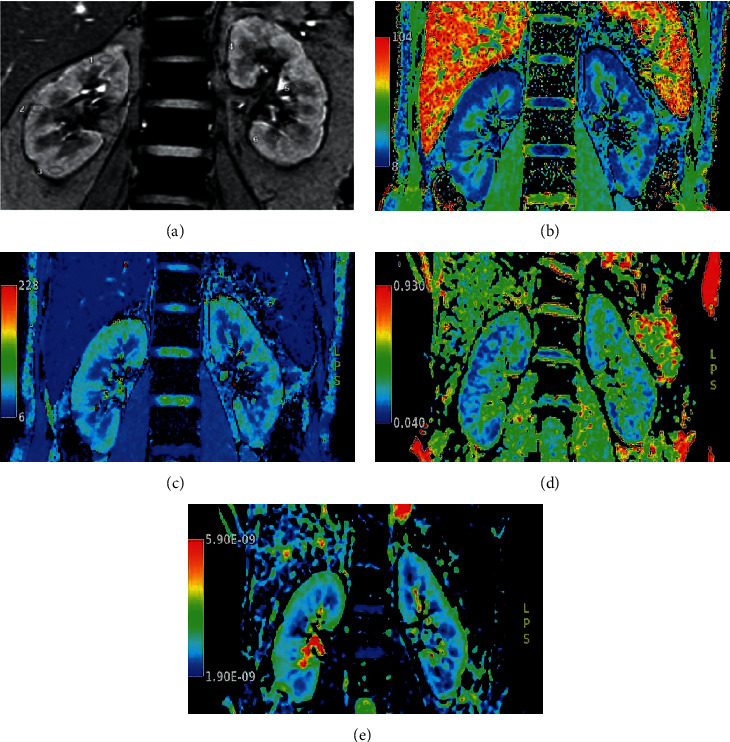
Examples of the positions of ROIs and pseudocolor pictures of BOLD and DTI images.Legends: An example of the measurement of the ROI on the coronal BOLD image (TE = 15.9 ms) in a 49-year-old woman (a). The ROIs were positioned in the cortex (1, 2, and 3) and medulla (4, 5, and 6) in the upper, middle, and lower parts of each kidney. The ROI had an area of 40–60 mm^2^. A pseudocolor figure of a participant in the DII group (male, 51 years old) was selected, where *b*/*c*/*d*/*e* correspond to the *R*2^*∗*^, *T*2^*∗*^, FA, and ADC images, respectively.

We measured the renal cortical *R*2^*∗*^ (*CR*2^*∗*^), medullary *R*2^*∗*^ (*MR*2^*∗*^), renal cortical FA (C.FA), renal medullary FA (M.FA), cortical average diffusion coefficient (C.ADC), and medullary average diffusion coefficient (M.ADC). All data were processed by two well-trained doctors independently with three years and six years of experience in imaging diagnosis, who were blinded to the population group before the measurement. We selected layers close to the renal hilum and manually drew regions of interest (ROIs) on BOLD and DTI images at the upper, middle, and lower poles of the cortex and the medulla of each kidney with ellipses of approximately 40–60 mm^2^. We tried to avoid renal blood vessels, edges, collecting systems, and cysts. Examples of pseudocolor images of the *R*2^*∗*^ map, FA map, and ADC map are displayed in Figure 1.

### 2.5. Laboratory Inspection

We collected biochemical data before or after the MRI examinations while excluding the effects of exercise, infection, fever, congestive heart failure, menstruation, significant hyperglycemia, and significant hypertension within 24 hours [18]. We calculated the eGFR based on the Chinese Modification of Diet in Renal Disease (MDRD). The improved formula for eGFR is as follows: eGFR (mL/min/1.73 m^2^) = 175 × (serum creatinine)^−1.154^ × (age)^−0.203^ × (0.742 female) [19, 20].

### 2.6. Statistical Analysis

Statistical analysis was performed using IBM SPSS Statistics 26.0, and the statistical significance was defined as *p* < 0.05. The parameters are expressed as the mean ± standard deviation (SD). One-way ANOVAs with the Bonferroni correction were used to test differences in *R*2^*∗*^, ADC, FA values among groups as well as *D* values (Δ, the measured medulla value-the corresponding cortical value) and MC ratios (MCR, the measured medulla value divided by the corresponding cortical value) of *R*2^*∗*^ and FA across groups. A Spearman correlation was also computed between the MRI parameters and clinical indices of renal function.

## 3. Result

### 3.1. General Clinical Characteristics of All Participants

The general clinical information and laboratory results are shown in (Table 1), including sex, age, hemoglobin (HGB), body mass index (BMI), eGFR, blood urea nitrogen (BUN), hemoglobin A1c (HbA1c), uAlb, UACR, and Cys C. There was no statistically significant difference in sex, age, or BMI among the HCs and the three diabetic subgroups. HbA1c in the three diabetes subgroups showed no difference. The mean eGFR of the DI group (115.25 ± 16.25 mL/min/1.73 m^2^) was slightly higher than that of the HC group (109.49 ± 9.70 mL/min/1.73 m^2^) (*p* > 0.05). HGB, SCr, BUN, uAlb, and Cys C were all significantly different among the three diabetes subgroups. The differences were mainly between the DI group and the DII/DIII group based on post-hoc multiple comparisons (*p* < 0.05).

Table 2 shows that there was no significant difference in sex, age, and clinical data between the DKD group (30 ml/min/1.73 m^2^ < eGFR < 90 ml/min/1.73 m^2^) and NDKD group.

Since renal puncture was an invasive method, most patients refused it when they were hospitalized. Among all the recruited subjects, 21 were definitely diagnosed with diabetes kidney disease and 10 more were diagnosed with NDKD by renal pathology. The specific pathological results and staging are shown in Table 3.

### 3.2. Comparison of *R*2^*∗*^, FA, and ADC of the Cortex and Medulla among the HC Group and the Three Diabetes Subgroups

Intraclass correlation coefficient (ICC) at a 95% confidence interval on values was analyzed, such as *CR*2^*∗*^ (ICC = 0.908, 95% CI 0.841–0.946), *MR*2^*∗*^ (ICC = 0.910, 95%CI 0.844–0.948), C.FA (ICC = 0.886, 95% CI 0.781–0.937), M. FA (ICC = 0.927, 95% CI 0.874–0.958), C.ADC (ICC = 0.929, 95% CI 0.861–0.961), and M. ADC (ICC = 0.898, 95% CI 0.828–0.940). All ICCs were all greater than 0.75. Data processed by the two doctors showed good consistency (*p* < 0.05).

**Figure 2:**
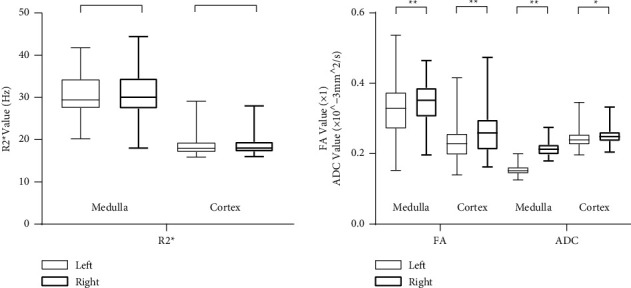
Comparison of the *R*2^*∗*^, ADC, and FA values between the right and left kidneys. Legends: Box plot comparing a total of six pairs including the *R*2^*∗*^ values, ADC values, and FA values between the left and right kidneys. ^*∗*^*p* < 0.01, ^*∗∗*^*p* < 0.001.

We performed paired-samples *t* test between the left and right kidneys (Figure 2). There was no difference in cortical and medullary *R*2^*∗*^ between the left and right kidneys (*p* > 0.05). However, FA and ADC values of right kidneys were significantly lower in both cortex (*t* = −6.054, *p* < 0.001; *t* = −0.530, *p* < 0.001) and medulla (*t* = −4.162, *p* < 0.001; *t* = −3.364, *p* < 0.01) than those of the left kidneys. Considering that the left kidney was most likely affected by the heart and blood vessels, we decided to individually utilize the DTI data of the right kidney.

*R*2^*∗*^, FA, and ADC values of the cortex were not significantly different among the HCs and the three diabetes subgroups (*p* > 0.05) (Table 4).

**Figure 3:**
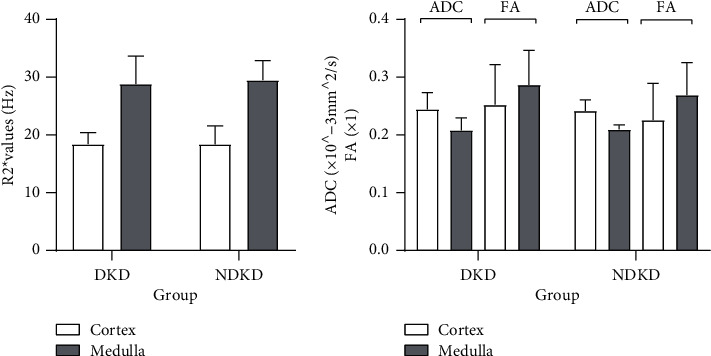
Comparisons of the *R*2^*∗*^ values, FA values, and ADC values of the cortex and medulla between the DKD and NDKD groups. Legends: Interleaved bars comparing the *R*2^*∗*^ values, FA values, and ADC values of the cortex and medulla between the DKD and NDKD groups. A *p* value < 0.05 was considered significant. No differences were found in the *R*2^*∗*^ values, FA values, or ADC values of the cortex and medulla between the DKD and NDKD groups.

Moreover, medullary *R*2^*∗*^ values in DI group were significantly higher than those in the HC group (*p* < 0.001). Medullary FA values in the HC group were higher than those in DI group (*p* = 0.005). Medullary ADC values were not significantly different among HCs, the DI, DII, and DIII group. We found no difference in *R*2^*∗*^, FA, and ADC values between DKD and NDKD (*p* > 0.05) (Figure 3).

**Figure 4:**
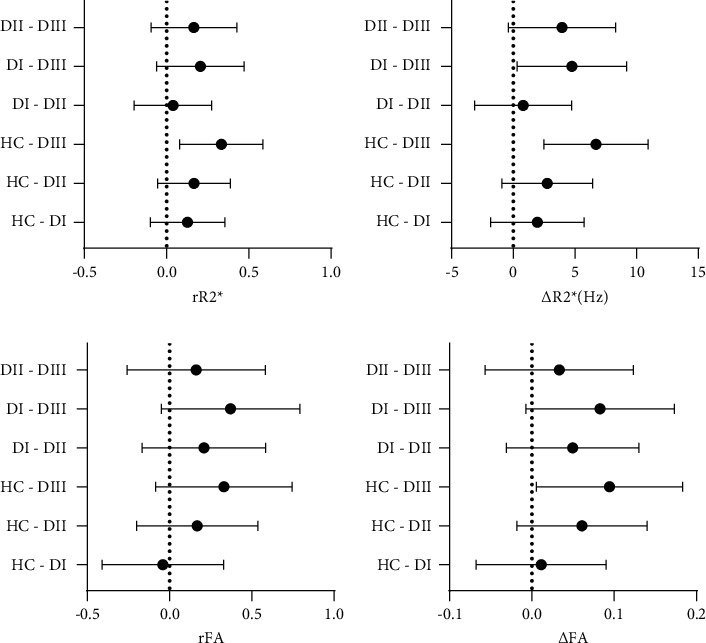
The D value and MCR evaluating the differences between the renal medulla and cortex among the groups. Legends: Plots of the 95% confidence intervals analyzing the *R*2^*∗*^ and FA values through D-values (e.g., Δ*R*2^*∗*^ = *MR*2^*∗*^ − *CR*2^*∗*^) and MCRs (e.g., *rR*2^*∗*^=*MR*2^*∗*^/*CR*2^*∗*^) of the renal medulla and cortex among the groups. The range of values that did not fall on the dotted line was considered to be significantly different.

To reduce individual differences, we calculated the D values (Δ) and MC ratios (*r*) between the renal medulla and the corresponding cortex (Figure 4). Relative to the stable differences between the cortex and medulla of the kidneys in the HC group, the changes in the DIII group implied an decreasing functional and structural differentiation of the cortex and medulla as renal function deteriorated.

### 3.3. Correlative Analysis

**Figure 5:**
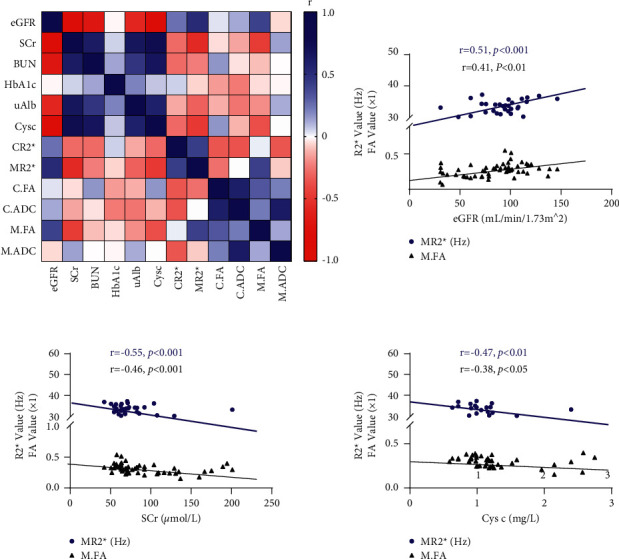
Correlations between clinical indexes and MRI parameters.Legends: Heatmap analyzing the correlation between the cortex and medulla *R*2^*∗*^, FA, and ADC values and the eGFR, BUN, HbA1c, uAlb, and Cys C levels of all the groups. The colormap was drawn with a double gradient. The *r* value ranges from −1 to 1. Blue represents the maximum value and red represents the minimum value. The linear correlations of clinical indexes with MRI data *MR*2^*∗*^/M.FA were also shown above. A *p* value < 0.05 was considered significant.

Spearman correlation analyzed the relationships between the cortical and medullary *R*2^*∗*^, FA values, and the eGFR, BUN, HbA1c, uAlb, and Cys C values among the DM patients (Figure 5). The eGFR had a moderate and positive correlation with the *MR*2^*∗*^ value and a positive but weak correlation with the M.FA (*p* < 0.001). In contrast, SCr was moderately and negatively correlated with *MR*2^*∗*^ and negatively but weakly correlated with the M.FA (*p* < 0.001). Cys C had a weak and negative correlation with the *MR*2^*∗*^ and M.FA (*p* < 0.01, *p* < 0.05). In addition, there was a correlation between SCr and the *CR*2^*∗*^ value, but the correlation was weak.

## 4. Discussion

The present study verified the (patho) physiological differences between the kidney cortex and medulla. Based on the trends in *R*2^*∗*^ and FA values, we can get a general idea of the changes in renal oxygenation levels, water molecular diffusion, and renal microstructure during the progression of diabetes to DKD. Our results revealed that the *R*2^*∗*^ and FA values in the renal medulla were higher than those in the cortex in all the groups, and the same result was also seen in previous studies [21–24]. This demonstrated that the renal medulla was in a state of “physiological hypoxia” and provided evidence of a regular arrangement of radiating medullary loops, collecting ducts, and tiny vessels. *R*2^*∗*^ and FA values in DKD patients showed significant differences from those in the HCs. The decline in FA was expected because changes in renal pathology, including glomerulosclerosis, interstitial fibrosis, tubular injury, and inflammatory cell infiltration, altered the anisotropy of well-aligned tubules [25, 26]. Notably, in our study, medullary *R*2^*∗*^ had a clear tendency to rise at the very early stage of DKD and rapidly decline once renal function was significantly impaired, which was same with the previous studies [27, 28].

The oxygenation level of the human kidney depends on the careful and coordinated physiological structure, and its coordination is mainly reflected in the balance between the oxygen supply and oxygen consumption of the kidneys. From a hemodynamic point of view, blood flow and the oxygen-carrying capacity of arterial blood are mainly responsible for the activities of supplying oxygen to the kidneys. Processes such as renal tubular reabsorption also utilize the oxygen transported to the kidneys. The blood flow of the renal cortex is significantly higher than the blood flow of the renal medulla, and the renal medulla is in a “physiological hypoxia state”. The difference in blood flow between the cortex and medulla helps maintain the osmotic pressure gradient, which is beneficial for the body to concentrate urine. The progression of kidney disease often changes in function earlier instead of that in structure. The level of renal oxygenation is a very potential and particularly suitable indicator for the early evaluation of DKD. At present, chronic hypoxia has been found in animal models of DKD, and its possible mechanism is related to factors such as hypoxia-inducible factors, cytokines and inflammatory mediators, and renal tubular epithelial cell trans differentiation. In animal models, improving chronic hypoxia can indeed delay the progression of chronic kidney disease to a certain extent [11].

BOLD-MRI, using deoxyhemoglobin as an endogenous contrast agent, relies on its paramagnetic properties to obtain images that are sensitive to local tissue oxygen concentration. The increase of deoxyhemoglobin content in the blood causes the inhomogeneity of the magnetic field in the surrounding local tissue, which leads to the rapid dephasing of protons in the tissue and thus the signal decreases on *T*2^*∗*^*WI* images. According to formulas (1) and (2),

(1) $$\begin{array}{c} SI\left(t\right)=SI_{0}e^{-t/qT2^{*}}, \end{array}$$

(2) $$\begin{array}{c} R2^{*}={1/T2}^{*}. \end{array}$$

The apparent relaxation rate *R*2^*∗*^ can be obtained by calculating the monoexponential curve fitting of the signal intensity of a series of *T*2^*∗*^*WI* images at different echo times to the TE time. The ratio of oxyhemoglobin to deoxyhemoglobin is related to the partial pressure of oxygen in the blood. The partial pressure of oxygen in the capillaries being in equilibrium with the surrounding tissue, the signal changes detected by BOLD-MRI can be interpreted as changes in the partial pressure of oxygen in the tissue. The decrease of *R*2^*∗*^ value indicates that the concentration of oxygenated hemoglobin increases in the local tissue, the proportion of deoxygenated hemoglobin decreases, and the local partial pressure of PaO_2_ increases. BOLD-MRI technology was first applied to the study of brain function, and its basis mainly depends on the close connection between hemodynamics and brain neural activity. Prasad et al. were the first scholars to apply BOLD-MRI to assess the level of renal oxygenation in humans. They also found that the medullary *R*2^*∗*^ value of patients with kidney disease showed a significant decreasing trend after using diuretics, suggesting that diuretics can improve renal hypoxia [29]. In this study, for the subjects recruited, we excluded those who used diuretics and those taking antihypertensive drugs containing diuretics in the past month to prevent interference errors to *R*2^*∗*^ values.

*R*2^*∗*^ is proportional to the content of deoxyhemoglobin, so an increase in *R*2^*∗*^ represents a decrease in PO_2_ and tissue hypoxia [30]. DM patients might experience renal medulla hypoxia, which is related to the highly increased oxygen consumption and less blood supply in the medulla, such as active reabsorption of excess sodium, glomerular hyperfiltration, and increased Na+/K+–ATPase activity in a hyperglycemic state [28, 31]. Significant elevation of *MR*2^*∗*^ was shown in DM patients without kidney disfunction in our study. Considering that these patients had higher HGB values and eGFR levels compared with healthy controls and DKD patients, the elevated eGFR and HGB seemed to verify that the kidney tissue was in a state of hypoxia compensation. At this stage, some diabetic patients may experience increased blood pressure, and it is recommended that blood pressure should not be controlled too low. The removal of the high filtration state will cause insufficient renal blood perfusion and accelerate renal damage [32]. Besides, *R*2^*∗*^ was also affected by other factors, such as hemodynamic and structural changes, red blood cell volume and the vascular volume fraction, vessel geometry, and applied pulse sequence parameters, including factors influencing the oxygen dissociation curve [33]. In the later stages of DKD, hypoxia indeed takes place in the medulla despite the decreased *R*2^*∗*^ in diabetic patients with a moderately decreased eGFR. It was inferred that the eGFR decreased and the tubular reabsorption decreased, along with the metabolic products of renal tubular epithelial cells, interstitial inflammatory cells, and endothelial cells that stimulate interstitial inflammation and fibrosis in the kidney, so that oxygen consumption was decreased [34]. The phenomenon of “hypoxic remission” of the medulla appeared. Additionally, there was no significant difference on *MR*2^*∗*^ between the DKD and NDKD, implying that diabetic patients with different etiologies of CKD were consistent in their changes in renal oxygenation levels.

DWI imaging can noninvasively evaluate the movement of water molecules in vivo. In recent years, the study of renal DWI under physiological or pathological conditions has gradually become a hot topic. Diffusion tensor imaging (DTI-MRI) is a technology developed based on the DWI technology. It was an MRI technique proposed in 1994 to analyze white matter fiber tracts. It reflects the tensor information of the diffusional motion of water molecules in vivo by applying diffusion-sensitive gradients in at least 6 noncollinear directions. So, the description of water molecular motion is more precise. DTI employs parameter values to reflect tissue diffusion characteristics. An FA value, ranging from 0 to 1, measures the proportion of the anisotropic component of the diffusion of water molecules in the overall diffusion tensor. The DTI postprocessing program of the workstation can correct the EPI distortion. The formula (3) required to generate the FA map is as follows. *λ*_1_, *λ*_2_, and *λ*_3_ are the eigenvalues of the diffusion tensor.

(3) $$\begin{array}{c} FA=\sqrt{\frac{{\left(\lambda_{1}-\lambda_{2}\right)}^{2}+{\left(\lambda_{1}-\lambda_{3}\right)}^{2}+{\left(\lambda_{2}-\lambda_{3}\right)}^{2}}{2{\left(\lambda_{1}+\lambda_{2}+\lambda_{3}\right)}^{2}}.} \end{array}$$

The larger the value, the more restrictive the tissue is to the direction of diffusion of water molecules. Compared with DWI, DTI-MRI can not only observe the restricted degree of the diffusion movement of water molecules but also show the differences in the directionality of the diffusion movement of water molecules and provide information about the changes in the ultrastructure of the tissue, helping evaluate the subtleties in the structure of the tissue. This makes it possible to apply DTI imaging to the evaluation of kidney-related physiological function and pathological states. Specifically, DTI can indirectly reflect microstructural changes of normal or damaged tissues by diffusion-related parameters, such as ADC and FA values. ADC focuses on the intensity or freedom, and FA focuses on the direction of water molecular diffusion. With respiratory triggering technology, the image quality of renal DTI is maintained better despite the influence of other soft tissue and the breathing movement of the native kidney. Currently, it can be widely used in the study of autologous kidneys and transplanted kidneys.

In general, cortical blood flow was abundant, and the radially arranged tubule structure in the medulla limited the diffusion of water molecules, so the ADC value was higher and the FA value was lower in the cortex [35]. In our study, the cortical and medullary ADCs did not differ significantly between HCs and the three diabetic subgroups. But diabetic patients had slightly lower cortical ADCs than compared with HCs, and DKDs with moderately decreased eGFR had higher medullary ADCs than the other groups. The reason relied on the possibility that diabetic patients had higher blood viscosity than HCs, and the disruption of the initially regularly arranged microstructure in the renal medulla could no longer well restrict the free diffusion of water molecules. Medullary FA decreased gradually with the decline in eGFR in diabetic patients since the kidneys in early-stage DKDs were likely to undergo microscopic changes such as tubular ectasia, tubular epithelial cell edema and necrosis, and interstitial inflammatory cell infiltration and proliferation, which destroyed the microstructures and inhibited the movement of water molecules [36, 37]. The ADC and FA results in Feng's DTI study were similar to our study. It seemed that FA had more potential than ADC in determining the diffusion of water molecules and the microstructural changes in the human kidneys [33]. However, in some DWI studies, the advantages of apparent diffusion coefficient (ADC) values were reflected. Wang et al. found that the cortical ADC value in the DKD murine model was higher than in the blank model [38]. Emre et al. revealed that ADC values of renal parenchyma could be used for early diagnosis and clinical staging of patients with kidney disease [39]. Xu et al. found a negative correlation between renal parenchymal ADC values and the degree of interstitial fibrosis [40].

Medullary *R*2^*∗*^ and FA had positive correlations with eGFR and negative correlations with SCr and Cys C, which is consistent with Lu L's study [41]. Serum Cys C is filtered through the glomerulus, entirely reabsorbed, catabolized, and metabolized by the proximal tubular epithelium. In addition, Cys C is not disturbed by inflammatory reactions, tumors, hemolysis, bilirubin, triglycerides, etc., and is also independent of sex, age, muscle mass, and diet. Cys C is a more sensitive indicator than SCr for the diagnosis of renal impairment. Mojiminyi et al. [42] suggested that Cys C is a more sensitive and practical indicator for detecting DKD.

The advantages of BOLD and DTI allow them to refrain from radiation and there is no need for an injection of a contrast agent. The renal cortex and medulla worked together and somewhat differently and maintained a clear functional and structural differentiation. D-value and MCR compared the difference between the cortex and medulla in an individual kidney rather than focusing only on the changes in medullary *R*2^*∗*^ and FA values. Compared with HCs and simple DM patients, statistically reduced cortical-medullary differentiation of the kidney was shown in DKD patients with microalbuminuria and massive albuminuria. With the improvement of technical methods and the continuous optimization of postprocessing analysis, DTI will surely show more special applications in scientific research and clinical practice. Its prospects are very broad, especially combined with IVIM technology and BOLD-fMRI in the field of nephrology.

### 4.1. Limitations of the Study

The sample size of this study is relatively small. The number of cases in the subgroups requires amplification. In addition, this study only investigated BOLD and DTI parameters in assessing the changes in renal oxygenation levels and microstructure. It did not fully elucidate the mechanisms of renal hypoxia or provide insight into the specific changes in the structure in DKD combined with the pathological basis. Alterations in microcirculatory perfusion could also affect the result. At present, there is no unified technical standard for BOLD-MRI to detect renal oxygenation at home or abroad. The normal value of the relaxation rate is related to different field strengths. In DTI, diffusion gradients could cause eddy currents, which have an effect on the final parameters. Combining with arterial spin labeling (ASL) and IVIM, BOLD and DTI may be useful for a more comprehensive assessment of renal functional status.

## 5. Conclusion

In conclusion, our study shows that BOLD and DTI are effective and safe ways to estimate renal function through the oxygenation level, water molecular movement, and microstructural alterations in patients with DKD and it proves that BOLD performs better than DTI. With renal function declines, alterations can be found including the firstly rising and then falling medullary *R*2^*∗*^, continuously declining medullary FA and apparent cortex-medullary differentiation in DKD patients.

## Figures and Tables

**Table 1:** Characteristics of the subjects.

Characteristic	HC (*n* = 20)	DI (*n* = 22)	DII (*n* = 22)	DIII (*n* = 18)	*F*	*p*
Sex (M/F)	9/11	12/10	14/8	12/6	—	0.510
Age (Y)	54.23 ± 14.69	54.17 ± 14.32	57.75 ± 13.56	58.13 ± 15.68	0.184	0.906
BMI (kg/m^2^)	22.49 ± 1.42	24.42 ± 4.60	25.89 ± 2.31	24.65 ± 3.04	2.570	0.068
HGB (g/L)	137.3 ± 8.1	145.9 ± 10.3	133.0 ± 11.3	110.6 ± 19.9	16.804	0.002
eGFR (mL/min/1.73 m^2^)	109.49 ± 9.70	115.25 ± 16.25	74.88 ± 7.43	44.44 ± 11.92	75.923	<0.001
Scr (*μ*mol/L)	58.74 ± 5.87	61.83 ± 9.29	84.96 ± 14.00	138.38 ± 45.31	18.114	<0.001
BUN (mmol/L)	5.05 ± 0.92	5.19 ± 0.98	6.92 ± 1.68	7.70 ± 3.14	2.764	0.012
Cys C (mg/L)	0.85 ± 0.18	0.89 ± 0.17	1.12 ± 0.31	1.91 ± 0.68	13.688	0.002
HbA1c (%)	N/A	6.43 ± 0.69	7.03 ± 1.68	6.27 ± 0.72	1.183	0.424
uALB (mg/L)	6.5 (2.8, 12.6)	9.8 (3.2,12.3)	150.0 (28.1, 171.5)	2817.7 (565.3, 3640.3)	11.425	0.038
UACR (mg/g)	12.2 (7.8, 15.3)	13.9 (8.7, 15.8)	58.5 (39.7, 62.5)	2742.0 (567.3, 3813.3)	28.402	<0.001

Legends: The results are expressed as the mean ± SD. If the normal distribution is not satisfied, the results are expressed by quartile method. The chi-square test was used to test the difference in sex in the four groups. ANOVA was used to test difference in the other characteristics among the abovementioned groups. A *p* < 0.05 was considered significant. BMI (body mass index), weight (kg)/height (m^2^); HGB, hemoglobin; eGFR, estimated glomerular filtration rate; SCr, serum creatinine; BUN, blood urea nitrogen; uALB, urinary microalbumin; Cys C, cystatin C; HbA1c, hemoglobin A1c; HC, healthy control group; DI, diabetes patients without albuminuria; DII group, diabetic kidney disease patients with microalbuminuria; DIII group, diabetic kidney disease patients with massive albuminuria.

**Table 2:** Characteristics of the DKD group vs. NDKD group.

Characteristic	DKD (*n* = 20)	NDKD (*n* = 10)	T	*P*
Sex (M/F)	14/6	8/2	—	0.559
Age (y)	57.30 ± 14.05	59.30 ± 10.10	−0.400	0.692
BMI (kg/m^2^)	25.43 ± 2.59	25.71 ± 3.03	−0.266	0.765
HGB (g/L)	123.6 ± 18.8	131.8 ± 23.3	−1.028	0.143
eGFR (mL/min/1.73 m^2^)	62.71 ± 17.85	59.52 ± 25.70	0.397	0.694
Scr (*μ*mol/L)	106.33 ± 39.88	121.20 ± 47.63	−0.903	0.374
BUN (mmol/L)	7.23 ± 2.33	9.53 ± 7.15	−1.325	0.196
Cys C (mg/L)	1.44 ± 0.62	2.41 ± 2.99	−1.020	0.333
HbA1c (%)	6.75 ± 1.43	6.77 ± 0.99	−0.153	0.880
uALB (mg/L)	1781.2 (1562.3, 1893.5)	1889.0 (1728.4, 2065.3)	−0.171	0.866
UACR (mg/g)	265.6 (243.4, 287.1)	280.4 (236.7, 310.6)	−0.893	0.799

Legends: An independent sample *t* test was used to compare the other characteristics in two groups. A *p* < 0.05 was considered significant. NDKD group, nondiabetic kidney disease patients; DKD group, diabetic kidney disease patients with matched eGFR.

**Table 3:** Staging of DKD and NDKD patients based on renal biopsy.

Stage of DKD	DKD (*n* = 21)	NDKD (*n* = 10)
Stage I: Glomerular hyperfiltration	*N* = 2	Diabetic patients with other nephropathy (membranous nephropathy, *N* = 2, hypertensive renal impairment *N* = 3, glomerulonephritis disease, *N* = 3, tubulointerstitial disease, *N* = 2)
Stage II: Silent stage	*N* = 4	
Stage III: Incipient nephrology	*N* = 12	
Stage IV: Overt nephrology	*N* = 3	

Legends: The final outcome for staging of patients with DKD took into account both albuminuria and pathological findings. None of the 21 DKD patients had other etiologies of CKD.

**Table 4:** Comparisons of *R*2^*∗*^ values, FA values, and ADC values of the cortex and medulla among HC and DM subgroups.

		HC	DI	DII	DIII	*p*	*p* ^*#*^	*p* ^&^	*p* ^*∗*^
Cortex	*R*2^*∗*^ ms^−1^	18.39 ± 0.82	19.41 ± 2.78	19.01 ± 2.03	17.50 ± 1.62	0.290	—	—	—
	FA	0.244 ± 0.062	0.231 ± 0.052	0.244 ± 0.068	0.266 ± 0.074	0.274	—	—	—
	ADC (×10^−3^ mm^2^/s)	0.255 ± 0.032	0.241 ± 0.022	0.244 ± 0.024	0.246 ± 0.036	0.583	—	—	—

Medulla	*R*2^*∗*^ ms^−1^	31.07 ± 2.11	33.12 ± 3.66	30.92 ± 4.46	25.46 ± 3.43	0.001	—	0.003	0.011
	FA	0.386 ± 0.080	0.322 ± 0.056	0.291 ± 0.055	0.280 ± 0.069	0.002	0.005	0.005	—
	ADC (×10^−3^ mm^2^/s)	0.205 ± 0.022	0.204 ± 0.023	0.202 ± 0.016	0.217 ± 0.027	0.498	—	—	—

Legends: The results are expressed as the mean ± SD. *R*2^*∗*^, rate of spin dephasing (Hz); FA, fractional anisotropy; ADC, average diffusion coefficient (×10^−3^ mm^2^/s). *p*: ANOVA was used to compare the values among all above groups, and a *p* value < 0.05 was considered significant. *p*^#^: Post-hoc paired comparisons between the HC group and the DII group; *p*^&^: Post-hoc paired comparisons between the HC group and the DIII group; *p*^*∗*^: Post-hoc paired comparisons between the DI and the DIII group.

## Data Availability

The data used to support the findings of this study are restricted by the data providers.

## Abbreviations

ADA: American Diabetes Association
BUN: Blood urea nitrogen
BMI: Body mass index
C.ADC: Cortical average diffusion coefficient
*CR*2^*∗*^: Cortical *R*2^*∗*^
Cys C: Cystatin C
DM: Diabetes mellitus
DKD: Diabetic kidney disease
eGFR: Estimated glomerular filtration rate
EPI: Echo-planar imaging
HbA1c: Hemoglobin A1
HC: Healthy control
HGB: Hemoglobin
ICC: Intraclass correlation analysis
KDIGO: Kidney Disease: Improving Global Outcomes
M.ADC: Medullary average diffusion coefficient
MDRD: Modification of Diet in Renal Disease
M.FA: Medullary FA
MFGRE: Multiple fast gradient recalled echo
*MR*2^*∗*^: Medullary *R*2^*∗*^
NDKD: Nondiabetic kidney disease
ROI: Regions of interest
SCr: Serum creatinine
SE: Spin-echo
UACR: Urinary albumin-creatinine ratio
uAlb: Urinary microalbumin.
